# Knowledge and Attitudes about Contraindications and Precautions to Vaccination among Healthcare Professionals Working in Vaccination Clinics in Ningbo, China: A Cross-Sectional Survey

**DOI:** 10.3390/vaccines12060632

**Published:** 2024-06-06

**Authors:** Lixia Ye, Qiuhong Mei, Pingping Li, Yueyi Feng, Xiaoqing Wu, Tianchi Yang

**Affiliations:** 1 Department of Immunization Program, Ningbo Municipal Center for Disease Prevention and Control, Ningbo 315010, China; nbcdcvaccination@163.com (L.Y.); ajmqh@163.com (Q.M.); yueyetupro@163.com (Y.F.); 2 Department of Immunization Program, Jiangbei District Center for Disease Prevention and Control, Ningbo 315021, China; lipingping0218@163.com; 3 School of Public Health, Ningbo University, Ningbo 315211, China; 15082864833@163.com

**Keywords:** vaccination, contraindications and precautions, healthcare professionals, knowledge, attitudes

## Abstract

Background: Healthcare professionals’ misjudgment of contraindications to vaccination can lead to unnecessary delays or missed vaccinations. It is essential to evaluate the knowledge and attitudes of healthcare professionals towards this issue. Methods: A two-phase cross-sectional study was conducted among healthcare professionals in vaccination clinics in Ningbo in 2022. The study data were collected using questionnaires evaluating the knowledge and attitudes of contraindications and precautions to vaccination. Knowledge scores were calculated and a cutoff of 75 was defined for adequate knowledge scores. Results: A total of 761 participants completed the questionnaire on attitudes. The majority of participants (86.20%) considered screening for vaccination contraindications to be the most important aspect of the vaccination administration process. A higher level of work stress was observed among full-time personnel engaged in this work. A total of 301 participants completed the questionnaire on relevant knowledge and practical experience. The median (IQR) total score was 75.00 (21.88). The lowest median score was observed for questions pertaining to disease diagnosis and classification (median: 40.00; IQR: 40.00). Regarding knowledge about vaccination contraindications, the scores for questions regarding national guidelines or vaccine package inserts (median: 85.71; IQR: 14.29) and guidelines from the WHO or ACIP (median: 100.00; IQR: 0.00) were higher than those derived from expert consensuses or literature findings (median: 71.43; IQR: 28.57) (*p* < 0.001). Higher scores were observed in the age group of 50–59 years, which included those who had received training twice or more times and those with relevant work experience. Conclusions: The knowledge of healthcare professionals working in vaccination clinics related to contraindications and precautions to vaccination is not sufficient, particularly regarding disease diagnosis and classification. Knowledge enhancement through repetitive skill training is required.

## 1. Introduction

Vaccines have been a highly effective strategy for reducing the morbidity and mortality associated with vaccine-preventable infectious diseases [1]. To date, a range of vaccines have been developed to prevent more than 20 life-threatening diseases, thereby enabling people of all ages to live longer and healthier lives [2]. According to the estimates of the World Health Organization (WHO), immunization currently prevents 3.5–5 million deaths annually from infectious diseases [2]. Since the establishment of the National Immunization Program (NIP), China has substantially reduced the burden of many vaccine-preventable diseases by sustaining high rates of NIP vaccine coverage that have exceeded 80% [3]. Nevertheless, some NIP vaccines have not yet achieved the target of 90% set by the Chinese government to achieve herd immunity and interrupt the transmission of vaccine-preventable diseases [4]. Furthermore, there is even a huge gap between the vaccination rates of NIP vaccines and those of non-NIP vaccines. For the majority of non-NIP vaccines, the coverage rates are less than 50% [3]. Despite the emphasis placed on immunization by healthcare systems for this high-risk population due to their increased susceptibility to developing serious negative outcomes from infectious diseases, the proportion of non-vaccination or delayed vaccination is much higher in this population than in the healthy population [5]. One of the main reasons for this phenomenon is that vaccination service providers in vaccination clinics refuse to immunize people with some medical conditions [6,7].

In China, vaccinations are administered in vaccination clinics, which are typically located in community healthcare centers. The vaccination process involves four steps: screening for contraindications and precautions to vaccination, registration, injection, and observation after vaccination in clinics for 30 min. The initial step in the administration process is for healthcare professionals to collect the vaccinee’s health condition and verify the presence of any diseases or conditions that could potentially lead to serious adverse events or decreased vaccine efficacy. Once this assessment has been completed, healthcare professionals must consider the risks and benefits of administering the vaccine and then provide advice on whether the vaccine can be administered in accordance with the vaccine package inserts, guidelines, and expert consensus on vaccines. As the most trusted advisors and influencers, healthcare professionals play a pivotal role in vaccination decisions. The false judgment of contraindications for vaccination may lead to unnecessary delays or missed vaccinations among the population with medical conditions [7,8,9,10]. However, there have been few studies examining how the screening for contraindications and precautions to vaccination are conducted by healthcare professionals in vaccination clinics in China.

In this research, we conducted a cross-sectional study among healthcare professionals in vaccination clinics in Ningbo City, which is an economically developed coastal city with a permanent population of 9.618 million located in the Zhejiang Province. This study aimed to assess the capacity of healthcare professionals in vaccination clinics to correctly identify contraindications to vaccination based on their level of knowledge and their attitudes towards this work in Ningbo City, China.

## 2. Materials and Methods

### 2.1. Study Population and Design

This cross-sectional study was conducted in two phases in Ningbo City. In the first phase, between February and March 2022, an initial survey of perceptions and attitudes about screening for contraindications and precautions to vaccination was conducted among the healthcare professionals in all 162 vaccination clinics in Ningbo. The calculated sample size was 96 healthcare professionals using the formula: $n={\left(\frac{z_{\alpha /2}CV}{\varepsilon }\right)}^{2}$, with a coefficient of variation of 0.50 [11], a confidence level of 95%, and a sample error of 10%. To obtain a representative sample for participant selection, 5 healthcare professionals from each vaccination clinic were invited to participate in the survey. Thus, a total of 810 healthcare professionals were invited. Actually, 761 healthcare professionals responded to this survey, and the response rate was 93.95%. In the second phase, the calculated sample size was 235 healthcare professionals using formula: $n=\frac{z_{\frac{\alpha }{2}\times }^{2}(1-p)}{\varepsilon^{2}p},$ considering an estimated rate of correct answers for knowledge about contraindications and precautions to vaccination of 62.07% [12], a confidence level of 95%, and a sample error of 10%. We conducted an investigation about the relevant knowledge and practical experience of contraindications and precautions to vaccination as a part of Ningbo’s annual vaccination training program during the period of 6–9 November 2022. A total of 367 healthcare professionals working in vaccination clinics in Ningbo who took part in this training program were invited to complete this investigation on their own within 30 min. Actually, 301 healthcare professionals completed this survey, and the response rate was 82.02%.

### 2.2. Questionnaire

For both surveys, self-administrated structured questionnaires were developed by the research team. The questionnaire on the perceptions and attitudes towards screening for contraindications and precautions to vaccination (Table S1) consisted of two sections: (1) basic information (age, educational background, occupational specialty, etc.) and (2) perceptions and attitudes toward screening for contraindications and precautions to vaccination, including familiarity, importance, and perception of this work. For the perception of this work, the respondents were asked to score on a scale of 0–5 for 5 items (stress, difficulty, working time, complexity of system operation, and satisfaction) on screening for contraindications and precautions to vaccination. The structural validity assessment of the questionnaire via a confirmatory factor analysis extracted factors that accounted for 0.67 of the variance, indicating substantial construct validity. The Cronbach’s coefficient of this questionnaire was 0.74, which indicated good internal consistency and reliability.

For the questionnaire on the relevant knowledge and practical experience regarding contraindications and precautions to vaccination, a literature review was conducted in order to identify plausible questions on the research topic. The questionnaire was developed using a range of sources, including vaccine package inserts, national guidelines from the Chinese immunization program [13], guidelines from the WHO [14] and the Advisory Committee on Immunization Practice (ACIP) [15], as well as a series of expert consensuses published in China [16] or literature findings for the vaccination of people with medical conditions. These sources were used to inform the questions on the knowledge and practical experience regrading contraindications and precautions to vaccination. Finally, a questionnaire comprising 36 items was formed, mainly containing the following fields of inquiry (Table S2): (1) basic information (age, relevant work experience, occupational specialty, and occasions of relevant training) and (2) knowledge and practical experience on the contraindications and precautions to vaccination (part 2, 32 items), mainly containing the following fields of inquiry: (1) knowledge of the contraindications and precautions to vaccination (part A, 17 items), including knowledge from national guidelines or vaccine package inserts (7 items), knowledge from guidelines from the WHO or ACIP (3 items), and knowledge from expert consensuses or literature findings (7 items); (2) knowledge of disease diagnoses and classifications (part B, 5 items); and (3) case scenarios on screening for contraindications and precautions to vaccination (part C, 10 items). In part 2, two options (“Yes” and “No”) were provided for each question. The structural validity assessment of the questionnaire, via a confirmatory factor analysis, extracted factors that accounted for 0.60 of the variance, indicating acceptable construct validity. The Cronbach’s coefficient of the questionnaire was 0.79, which indicated good internal consistency and reliability. The online link to the questionnaires was sent to the participants with an explanation of the questionnaires. The integrity checking of the questionnaire was automatically completed on the online questionnaire platform.

### 2.3. Statistical Analyses

A normality omnibus test based on skewness and kurtosis coefficients was conducted to assess the descriptive statistics of the variables related to perceptions and attitudes. Due to the non-normal distribution of the variables, descriptive statistics of the variables related to perceptions and attitudes were conducted (median, interquartile range (IQR), frequency, and percentage). Differences in the variables of perception between full-time personnel and other personnel were analyzed using the Mann–Whitney U test. For the knowledge and practical experience regarding the contraindications and precautions to vaccination, the answers to each item were classified as correct or incorrect. The frequencies and percentages of the correct and incorrect answers for each statement were calculated. The total score was calculated using the following formula: score = $\frac{Number\;of\;correct\;answers\;}{Number\;of\;Items\;}\times 100$ (which ranged from 0 to 100). The scores from each subgroup were calculated using the following formula: score_subgroup_ = $\frac{Number\;of\;correct\;answers\;in\;subgroup}{Number\;of\;Items\;in\;subgroup\;}\times 100$, which ranged from 0 to 100. Higher scores indicated better knowledge. Descriptive statistics of the scores were calculated (median, IQR). The median total score (75) was employed as the cut-off value for the knowledge and practical experience regarding the contraindications and precautions to vaccination. A score of ≥75 was defined as adequate knowledge, while a score of <75 was defined as inadequate knowledge. To determine the distribution of the scores, skewness and kurtosis coefficients were applied, and it was observed that the scores were not normally distributed. Thus, the Mann–Whitney U test was used to compare the scores between independent paired groups, and the Kruskal–Wallis analysis was used to compare scores among more than two groups. A two-tailed *p*-value of <0.05 was considered statistically significant. Statistical analyses were performed using Stata 17.0 (Stata Corp, College Station, TX, USA).

## 3. Results

### 3.1. Basic Characteristics of the Participants

A total of 761 healthcare professionals working in vaccination clinics completed the questionnaire on their perceptions and attitudes about screening for contraindications and precautions to vaccination. Of these participants, the majority were from the 30–39 (45.60%) and 40–49 (30.62%) age groups, had 10–19 years of job experience (47.17%), held bachelor’s degree or above (82.26%), specialized in nursing (53.09%) or public health (25.62%), and had an intermediate (44.81%) or primary (40.34%) professional title. A total of 259 (34.03%) healthcare professionals were full-time personnel for screening for contraindications and precautions to vaccination. A total of 724 individuals (95.14%) received training in contraindications and precautions to vaccination. The details are presented in Table S3.

For the investigation on the relevant knowledge and practical experience on the contraindications and precautions to vaccination, a total of 301 healthcare professionals completed the questionnaire, of which the majority were also from the 30–39 (46.84%) and 40–49 (26.58%) age groups and specialized in nursing (47.84%) or public health (42.52%). A total of 250 (83.06%) participants reported having work experience in screening for contraindications and precautions to vaccination. Additionally, 285 (94.68%) individuals received training in contraindications and precautions to vaccination. The details are shown in Table S4.

### 3.2. Perceptions and Attitudes about Screening for Contraindications and Precautions to Vaccination

Of the 761 participants, 457 (60.05%) considered themselves familiar with screening for contraindications and precautions to vaccination, while 185 (24.31%) believed they were very familiar with this work. Moreover, 656 (86.20%) healthcare professionals identified screening for contraindications and precautions to vaccination as the most important work among the four steps of vaccination administration. With regard to the perception of this work, the median (interquartile range) scores for work stress, work difficulty, screening time, complexity of system operation, and work satisfaction were 4 (2), 3 (2), 3 (1), 3 (2), and 3 (1), respectively. The Mann–Whitney U test demonstrated that there were statistically significant differences between the full-time personnel engaged in screening for contraindications and precautions to vaccination and the other healthcare professionals in work stress and screening time, with the former group exhibiting a higher level of work stress and requiring a longer screening time for each case (Table 1).

### 3.3. Knowledge and Practical Experience on Contraindications and Precautions to Vaccination

#### 3.3.1. Total Score

The median total score was 75.00 (21.88), with 154 (51.16%) healthcare professionals demonstrating an adequate level of knowledge, while 147 (48.84%) participants did not reach this level. As illustrated in Table 2, the total score was the highest in the 50–59 age group (median: 84.38; IQR: 15.63), and the lowest total score was observed in the 18–29 age group (median: 68.75; IQR: 15.63) (*p* = 0.012). Those with work experience in screening for contraindications and precautions to vaccination demonstrated a higher total score (median: 75.00; IQR: 21.87) than those without it (median: 71.88; IQR: 25.00) (*p* = 0.019). Furthermore, a higher total score was observed in healthcare professionals who received relevant training twice or more (median: 75.00; IQR: 25.00) (*p* = 0.005) in comparison with those who took part in relevant training once or never (median: 71.88; IQR: 15.63). The scores of the three parts of the questionnaire were found to be significantly different (*p* < 0.001). The score from the questions on the contraindications and precautions to vaccination (part A) was the highest (median: 88.24; IQR: 17.65), while the score from the questions on disease diagnoses and classifications (part B) was the lowest (median: 40; IQR: 40).

#### 3.3.2. Knowledge of Contraindications and Precautions to Vaccination

**Figure 1:**
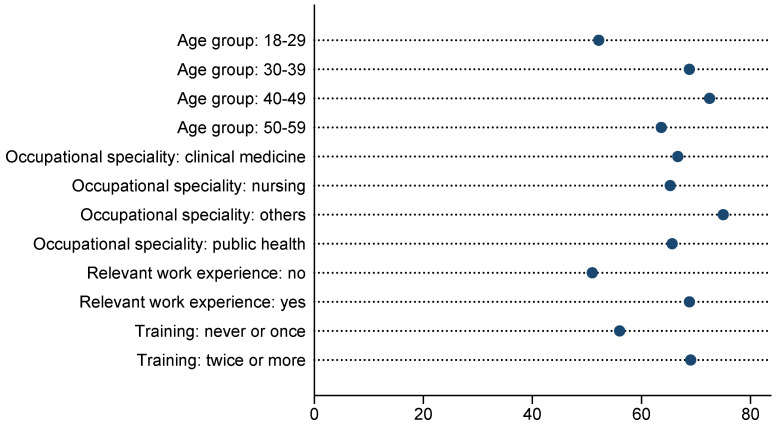
The proportion of correct responses to questions regarding egg allergy stratified according to the subgroups (%).

The results of part A indicate that 240 (79.73%) healthcare professionals reached an adequate level of knowledge, while 61 (20.27%) did not. The results indicated that the scores of the 50–59 age subgroup (median: 94.12; IQR: 11.77), the subgroup with relevant work experience (median: 88.24; IQR: 17.65), and the subgroup that received training twice or more (median: 88.24; IQR: 17.65) were higher than those of the other subgroups (Table 2). As shown in Table 2, the median scores on the questions on knowledge from the national guidelines for the national immunization program or vaccine package inserts (median: 85.71; IQR: 14.29) and the guidelines from the WHO or ACIP (median: 100.00; IQR: 0.00) were higher than the median score on the questions on knowledge from expert consensuses or literature findings (median: 71.43; IQR: 28.57) (*p* < 0.001). In addition, there were no significant differences in the scores on the questions on knowledge from the national guidelines for the national immunization program or vaccine package inserts and scores from the guidelines from the WHO or ACIP between each subgroup. Moreover, 84.39% and 93.69% of the participants reached an adequate level of knowledge about the national guidelines or vaccine package inserts and about the guidelines from the WHO or ACIP, respectively. In contrast, only 47.18% demonstrated an adequate level of knowledge regarding questions pertaining to expert consensuses or literature findings. The correct answer rates of all the questions in this part are presented in Table 3. The correct answer rates ranged from 38.87% to 99.34%. In this part of the questions, the lowest correct answer rate (38.87%) was seen in the question about contraindications to the vaccination of patients with complement deficiency disease, which is a group of diseases characterized by immunodeficiency. The correct answer rates were also low in the question about contraindications to the vaccination of patients recovered from perianal abscesses (62.46%), patients with hemolytic anemia (71.43%), and patients with chronic liver disease (67.44%). Additionally, 34.22% of healthcare professionals were unaware that an egg allergy was not a contraindication for measles-containing vaccines. In contrast to the responses observed in other questions within this part, the proportion of correct answers regarding egg allergy was found to be significantly lower among individuals in the 50–59 age group compared to those in the 30–39 and 40–49 age groups (*p* = 0.049) (Figure 1).

#### 3.3.3. Knowledge of Disease Diagnoses and Classifications

As for the knowledge of disease diagnoses and classifications, only 71 (23.59%) healthcare professionals reached an adequate knowledge level, while 230 (76.41%) did not. The scores of all the subgroups were low (Table 2), indicating a poor level of knowledge among healthcare professionals in vaccination clinics regarding disease diagnoses and classifications. The correct answer rates of the questions ranged from 28.24% to 65.78%. The lowest correct answer rate (28.24%) was observed in the question about the category of primary immunodeficiency. Furthermore, 56.48% and 57.81% of participants did not know the definitions of simple febrile seizures and stable seizure control, respectively. Additionally, 34.22% of participants had difficulty in correctly judging the normality of cardiac function based on ejection fraction. A total of 51.16% of participants provided an accurate response to the question regarding the classification of autoimmune diseases.

#### 3.3.4. Responses to Case Scenarios on Screening for Contraindications and Precautions to Vaccination

The correct answer rate for each question in the 10 case scenarios on screening for contraindications and precautions to vaccination ranged from 53.49% to 87.38% (Table 4). In the case scenarios of common diseases, such as asthma, food allergies, and febrile convulsions, the correct answer rates were high. The lowest correct answer rate was observed in two case scenarios involving children born to HIV-infected mothers (53.49% and 57.48%). Additionally, the correct answer rates were also low in some complex case scenarios, such as contraindications and precautions to vaccination in children with severe combined immunodeficiency following stem cell transplantation, patients with kidney disease, and those receiving immunosuppressive agents (Table 4). The score for this part (Figure 1) indicated that 144 (47.84%) healthcare professionals achieved an adequate level, while 157 (52.16%) did not. Notably, 81.82% of participants reached the adequate level in the 50–59 age group, which was considerably higher than in other age groups. Among healthcare professionals who did not previously participate in relevant training or only received one training session, only 29.33% achieved the adequate level. In contrast, the percentage of those who achieved the adequate level increased to 53.98% among those who had received two or more training sessions (*p* < 0.001).

## 4. Discussion

In China, instances of missed opportunities or unnecessary delays in vaccinations are more prevalent than anticipated, particularly among individuals with pre-existing medical conditions. Studies conducted in Zhejiang China [6,17] revealed that most children with medical conditions, such as congenital heart disease and seizures, were delayed or even contraindicated in vaccination. However, more than 80% of them were recommended by experts to be vaccinated on the nationally recommended schedule, and no serious side effects occurred after vaccination. The major reason for this discrepancy is that providers in the community health center overestimated the contraindications to vaccination and refused to administer vaccines for the sake of safety. Consequently, the attitudes and knowledge of contraindications and precautions among vaccination providers potentially influence reasonable vaccination. This study assessed different aspects related to the attitudes, knowledge, and practices of healthcare professionals in the vaccination clinics that provided vaccination services to the public regarding contraindications and precautions to vaccination.

In China, considering they may play different roles in their daily work, all healthcare professionals working in vaccination clinics are required to possess the ability to perform all work tasks, including contraindication screening, registration, injection, and medical emergency management. In this study, although only 34.03% of the healthcare professionals in the vaccination clinics were registered as full-time personnel for contraindication screening, 83.06% had this work experience. The investigation revealed that the median total score for knowledge and practical experience on contraindications and precautions to vaccination was 75, and the correct answer rates of each question ranged from 28.24% to 99.34%. This indicated that there were still knowledge gaps among healthcare professionals in vaccination clinics, in accordance with the findings of studies conducted in Beijing [12] and Shanghai [18]. The same knowledge gaps for contraindications and precautions to vaccination were also observed in healthcare professionals in other countries. A study conducted in Europe [7] demonstrated that 21.9% of responses to case scenarios in healthcare providers indicated false vaccine contraindications. Another study conducted in Albania [19] revealed that only 13% of nurses in vaccination clinics answered at least six questions correctly among ten questions regarding knowledge about childhood vaccine contraindications. In the part on the contraindications and precautions to vaccination, the results showed a comprehensive understanding of the national guidelines for the national immunization program or vaccine package inserts and the guidelines from the WHO or ACIP but an inadequate grasp of the expert consensus among all the healthcare professionals in vaccination clinics. The inclination towards official guidelines is primarily due to the fact that expert consensus statements are not recognized as official documents under the Vaccine Administration Law of the People’s Republic of China (PRC). In 2021, the National Health Commission of the PRC updated the national guidelines for the national immunization program and incorporated a new section on “vaccination recommendations for children with common special health conditions”. This section included prevalent childhood medical conditions, such as prematurity, low birth weight, allergic predisposition, immune system dysfunction, congenital diseases, and congenital infections. In addition, The Regional CDCs have placed a greater emphasis on the necessity of pre-employment education and assessments for healthcare professionals working in vaccination clinics, with a particular focus on the importance of knowledge of official documents and guidelines. Therefore, the results of this study demonstrated that the accuracy of responses to questions pertaining to national guidelines exceeded 95%, with the exception of the query regarding patients with complement deficiency. Despite the explicit mention of this guideline, only 38.87% of the healthcare professionals were aware that attenuated live vaccines were not contraindicated in patients with complement deficiency, while 61.13% continued to view complement deficiency as a contraindication to vaccination. This misunderstanding can be attributed to a lack of awareness among healthcare professionals working in vaccination clinics regarding complement deficiencies, which are a group of diseases with a low prevalence belonging to the category of primary immunodeficiencies [20]. In this study, the correct answer rates of the questions on knowledge regarding disease diagnoses and classifications, which ranged from 28.24% to 65.78%, revealed knowledge gaps in disease diagnoses and classifications among healthcare professionals in vaccination clinics regardless of their specialty, further confirming this view. We also found that training did not result in a notable enhancement in this area of knowledge. This highlights the necessity for the inclusion of knowledge about disease diagnoses and classifications in the training programs for healthcare workers in vaccination clinics in Ningbo. In order to resolve this issue, it is recommended that a multidisciplinary consultation involving specialist clinicians should be implemented in screening for contraindications and precautions to vaccination in populations with medical conditions [21,22]. Furthermore, it is necessary to incorporate relevant courses into the training programs for healthcare professionals in vaccination clinics.

Although this study revealed a generally inadequate level of knowledge on the expert consensus among healthcare professionals in vaccination clinics, it is worth noting that still, 47.18% of the healthcare professionals reached an adequate level of knowledge. The primary rationale for this is that Ningbo CDC reviewed the expert consensus on vaccinating children with special medical conditions in China and subsequently issued a vaccination recommendations document in 2019 for healthcare professionals to implement. A previous study [23] identified the need for detailed official vaccination recommendations that carry authority among healthcare professionals as the most pressing issue in the field of vaccination services for children with medical conditions. While several relevant expert consensuses have been developed, it is crucial to establish clear and concise guidelines to ensure proper care. Meanwhile, training programs based on the recommendation document of Ningbo CDC were held in 2019 and 2020. The results of this study indicated that a significant improvement in knowledge of the expert consensus was observed in those who received training more than twice. Furthermore, the responses to case scenarios on screening for contraindications and precautions to vaccination demonstrated a similar improvement, suggesting that repetitive skill training can effectively enhance healthcare professionals’ knowledge and practice skills.

Another issue that merits consideration is the potential for confusion among healthcare professionals in vaccination clinics, particularly senior healthcare professionals, due to changes in vaccination contraindications. Despite the early evidence indicating that children with an egg allergy could be administered measles-containing vaccines [24,25], egg allergy was still considered a contraindication in vaccine package inserts in China until 2012 [8]. The results of our study indicated that, after the update in vaccine package inserts, 34.22% of healthcare professionals in vaccination clinics were unaware that egg allergy was no longer a contraindication for measles-containing vaccines. Although this issue has been highlighted during our annual training session, the awareness of this among senior healthcare professionals is lower than that of other age groups. This is in contrast to the awareness of other vaccine contraindications. A higher proportion of incorrect contraindication judgments among egg allergy patients was also reported in other countries, such as Ecuador [9]. Additionally, discrepancies between vaccine package inserts and official recommendations also contribute to confusion among healthcare professionals, impeding the vaccination of individuals with medical conditions [23]. The technical guidelines for seasonal influenza vaccination in China (2023–2024) [26] do not consider egg allergy as a contraindication to influenza vaccination, whereas some influenza vaccine package inserts still list it as a contraindication. Consequently, vaccine package inserts must be updated in a timely manner in accordance with the latest research evidence. Furthermore, a well-coordinated immunization policy is essential to guide the vaccination practice for potentially off-label vaccine recommendations.

The case scenarios on screening for contraindications and precautions to vaccination demonstrated that healthcare professionals possessed a comprehensive understanding of the contraindications of common diseases, such as asthma, food allergies, and febrile convulsions. This is a result of encountering such situations repeatedly in daily practice, which has consolidated their knowledge. In some case scenarios that are rarely encountered in vaccination clinics, such as children born to HIV-infected mothers, the correct response rate is significantly lower than for other scenarios. In addition, the lower correct response to complex case scenarios indicated that there were still obstacles between the acquisition of knowledge and its practical application.

In examining healthcare professionals’ attitudes towards screening for vaccine contraindications, our findings indicated that 86.20% of healthcare professionals in vaccination clinics considered this to be the most important aspect of the vaccination administration process, as any misjudgments made by healthcare professionals could result in missed opportunities or delays in children with medical conditions receiving the recommended vaccinations. Higher levels of work stress were observed among full-time personnel engaged in this work compared to other healthcare professionals. On the one hand, this stress may be linked to the mistrust of vaccine recipients toward healthcare professionals in vaccination clinics [27] and the fear of a possible increased risk of medical disputes due to adverse events from vaccination [11]. A previous study in China [28] showed that parents of children with medical conditions preferred to receive the vaccination assessment results from higher-level hospitals rather than community hospitals. On the other hand, this work necessitates a comprehensive understanding of a multitude of medical conditions, as well as contraindications and precautions to vaccination. This appears to be a significant challenge for healthcare professionals in vaccination clinics [11,16] as evidenced by the fact that the majority of them have not received clinical skill training. Furthermore, our findings indicated that full-time personnel responsible for contraindication and precaution screening were willing to dedicate more time to each case, suggesting that they may be more thorough in their investigation of the patient’s health status and more cautious in their assessment. Nevertheless, due to the considerable number of daily vaccination services, there was insufficient time to comprehensively enquire about the health status of the vaccine recipients [27].

There were limitations in the present study. Firstly, this study evaluated the ability of healthcare professionals working in vaccination clinics to correctly assess contraindications to vaccination only based on knowledge and responses to the case scenarios of screening for contraindications and precautions to vaccination, not the actual practice of vaccine contraindication screening in vaccination clinics. Consequently, future research should seek to ascertain the frequency with which misjudgments of vaccine contraindications occur in routine practice in vaccination clinics. Secondly, this study was conducted in two phases. In the investigation of the relevant knowledge and practical experience of contraindications and precautions to vaccination, in order to ensure that all participants completed the questionnaire independently within a limited time as in daily work scenarios, we selected the healthcare professionals who took part in Ningbo’s annual vaccination training program in 2022 as the study population. This non-probabilistic opportunistic sample may have resulted in selection bias. Furthermore, the investigation was limited to the healthcare professionals working in the vaccination clinics, with no consideration for other medical professionals (such as clinical specialists) and the recipients themselves or their guardians, whose input might influence the assessment of contraindications to vaccination. Thirdly, the sample size of this study was relatively limited, comprising only vaccination personnel in Ningbo. It is important to note that, in view of the considerable regional disparities in China, further studies should be conducted in different regions. Therefore, future research should endeavor to assess the opinions and perspectives of these vital stakeholders in this field.

## 5. Conclusions

In conclusion, this study revealed that the knowledge of healthcare professionals working in vaccination clinics in Ningbo related to contraindications and precautions to vaccination was not sufficient, particularly with regard to their understanding of disease diagnoses and classifications. Changes in vaccination contraindications and discrepancies between vaccine package inserts and official recommendations caused confusion among healthcare professionals. They require repetitive skill training to effectively enhance relevant knowledge and practical skills.

## Figures and Tables

**Table 1:** Scores on the perceptions and attitudes of healthcare professionals in vaccination clinics regarding screening for contraindications to vaccination (median (IQR), *N* = 761).

Items	Total	Full-Time Personnel for Screening ^a^	Other Personnel ^a^	z ^b^	*p*
Degree of work stress	4 (2)	4 (2)	4 (2)	−3.22	0.001
Degree of work difficulty	3 (2)	4 (2)	3 (1)	−1.24	0.215
Time of screening for one case	3 (1)	4 (2)	3 (1)	−2.25	0.024
Complexity of system operation	3 (2)	3 (2)	4 (2)	−0.15	0.881
Degree of work satisfaction	3 (1)	3 (1)	3 (1)	0.22	0.824

^a^ Full-time personnel for screening refers to the healthcare professionals who were engaged in screening for contraindications to vaccination full time at the time of the investigation, and other personnel refers to the healthcare professionals who were engaged in registration, injection, and medical emergency management. ^b^ The Mann–Whitney U test was employed for this analysis.

**Table 2:** Scores from the questions on relevant knowledge and practical experience on the contraindications and precautions to vaccination (median (IQR), *N* = 301).

Characteristic	Scores from Part A (Knowledge of Vaccine Contraindications and Precautions)				Score from Part B (Knowledge of Disease Diagnoses and Classifications)	Score from Part C (Case Scenario)	Total Score
	Subtotal	National Guidelines or SPC	Guidelines from the WHO or ACIP	Expert Consensuses or Literature Findings			
Total	88.24 (17.65)	85.71 (14.29)	100.00 (0.00)	71.43 (28.57)	40.00 (40.00)	70.00 (30.00)	75.00 (21.88)
Age							
18–29	82.35 (17.65)	85.71 (14.29)	100.00 (0.00)	71.43 (28.57)	40.00 (40.00)	70.00 (30.00)	68.75 (15.63)
30–39	88.24 (17.65)	85.71 (14.29)	100.00 (0.00)	85.71 (28.57)	40.00 (40.00)	80.00 (30.00)	75.00 (25.00)
40–49	88.24 (17.65)	85.71 (14.29)	100.00 (0.00)	71.43 (28.57)	40.00 (50.00)	70.00 (30.00)	75.00 (20.32)
50–59	94.12 (11.77)	100.00 (14.29)	100.00 (0.00)	85.71 (28.57)	60.00 (60.00)	90.00 (10.00)	84.38 (15.63)
	*p* = 0.007	*p* = 0.251	*p* = 0.054	*p* = 0.010	*p* = 0.697	*p* = 0.048	*p* = 0.012
Work experience in vaccine contraindication assessments							
Yes	88.24 (17.65)	85.71 (14.29)	100.00 (0.00)	78.57 (28.57)	40.00 (40.00)	70.00 (30.00)	75.00 (21.87)
No	76.47 (17.65)	85.71 (14.29)	100.00 (0.00)	71.43 (42.85)	40.00 (40.00)	70.00 (40.00)	71.88 (25.00)
	*p* = 0.009	*p* = 0.325	*p* = 0.270	*p* = 0.005	*p* = 0.477	*p* = 0.063	*p* = 0.019
Occupational specialty							
Clinical medicine	88.24 (5.89)	85.71 (14.29)	100.00 (0.00)	85.71 (14.28)	40.00 (40.00)	70.00 (30.00)	75.00 (21.87)
Public health	88.24 (17.65)	85.71 (14.29)	100.00 (0.00)	71.43 (28.57)	40.00 (50.00)	75.00 (30.00)	75.00 (21.88)
Nursing	82.35 (17.65)	85.71 (14.29)	100.00 (0.00)	71.43 (28.57)	40.00 (40.00)	70.00 (30.00)	71.88 (18.75)
Others	91.18 (14.71)	92.86 (14.29)	100.00 (0.00)	85.71 (14.28)	50.00 (40.00)	85.00 (30.00)	84.38 (23.44)
	*p* = 0.283	*p* = 0.838	*p* = 0.567	*p* = 0.163	*p* = 0.641	*p* = 0.936	*p* = 0.648
Occasions of relevant training							
Never or once	82.35 (17.65)	85.71 (14.29)	100.00 (0.00)	71.43 (28.57)	40.00 (60.00)	60.00 (30.00)	75.00 (25.00)
Twice or more	88.24 (17.65)	85.71 (14.29)	100.00 (0.00)	85.71 (28.57)	40.00 (20.00)	80.00 (30.00)	71.88 (15.63)
	*p* = 0.008	*p* = 0.280	*p* = 0.068	*p* = 0.010	*p* = 0.993	*p* < 0.001	*p* = 0.005

**Table 3:** Responses of healthcare professionals to knowledge about the contraindications and precautions to vaccination (*N* = 301).

Source of Knowledge	Statement	Theme	Correct n(%)	Incorrect n(%)
**Part A Knowledge of the contraindications and precautions to vaccination**				
National guidelines for the national immunization program or vaccine package inserts	Q1. The vaccine is contraindicated in children who are allergic to its components.	Allergy	299 (99.34)	2 (0.66)
	Q2. Vaccinations are not contraindicated in people with simple febrile seizures.	Simple febrile seizures	292 (97.01)	9 (2.99)
	Q3. Cerebral palsy is not a contraindication for vaccination.	Cerebral palsy	283 (94.02)	18 (5.98)
	Q4. Children with physiological jaundice or breast milk jaundice can get vaccinated if they are in good health.	Jaundice	286 (95.02)	15 (4.98)
	Q5. Asymptomatic children with congenital heart disease and preserved cardiac function can be vaccinated by all types of vaccines.	Congenital heart disease	296 (98.34)	5 (1.66)
	Q6. Attenuated live vaccines are not contraindicated in patients with complement deficiency.	Complement deficiency	117 (38.87)	184 (61.13)
	Q7. Children with autoimmune diseases can be vaccinated with inactivated vaccines during their remission phase.	Autoimmune diseases	287 (95.35)	14 (4.65)
Guidelines from the WHO or ACIP	Q8. Children with local reactions such as redness, swelling, heat and pain after vaccination are not contraindicated with this kind of vaccine.	General reaction to vaccination	294 (97.67)	7 (2.33)
	Q9. Children with fever after vaccination are not contraindicated with this vaccine.	Fever	294 (97.67)	7 (2.33)
	Q10. Vaccinations are not contraindicated in people with penicillin allergy.	Penicillin allergy	294 (97.67)	7 (2.33)
Expert consensuses or literature findings	Q11. Children who have recovered from perianal abscess can be vaccinated with live attenuated polio vaccine.	Perianal abscess	188 (62.46)	113 (37.54)
	Q12. Individuals with mild to moderate iron deficiency anemia, in the absence of other symptoms, are eligible for vaccination.	Iron deficiency anemia	274 (91.03)	27 (8.97)
	Q13. Children with mild to moderate hemolytic anemia, who do not exhibit acute hemolysis, are eligible for vaccination.	Hemolytic anemia	215 (71.43)	86 (28.57)
	Q14. Egg allergy is not a contraindication for measles-containing vaccines.	Egg allergy	198 (65.78)	103 (34.22)
	Q15. Patients with mild to moderate hyperbilirubinemia in the context of chronic liver disease can be vaccinated by all types of vaccines.	Chronic liver disease	203 (67.44)	98 (32.56)
	Q16. Children treated with immunosuppressants can be vaccinated with inactivated vaccines.	Immunosuppressant	232 (77.08)	69 (22.92)
	Q17. Patients with liver cirrhosis can be vaccinated with inactivated vaccines.	Liver cirrhosis	230 (76.41)	71 (23.59)
**Part B Knowledge of disease diagnosis and classification**				
Expert consensuses or literature findings	Q18. Simple febrile seizures are characterized by generalized seizures lasting less than 15 min, which occur once during the course of a fever.	Simple febrile seizures	131 (43.52)	170 (56.48)
	Q19. Stable seizure control refers to epilepsy patients who have been seizure-free for at least 6 months, regardless of their use of antiepileptic drugs.	Stable seizure control	127 (42.19)	174 (57.81)
	Q20. The category of primary immunodeficiency includes chronic granulomatous disease, combined immunodeficiency, and immune dysregulation disease; however, thrombocytopenic purpura does not fall within this classification.	Category of primary immunodeficiency	85 (28.24)	216 (71.76)
	Q21. Type 1 diabetes, dermatomyositis, Sjogren’s syndrome, and ankylosing spondylitis are all classified as autoimmune diseases.	Category of autoimmune diseases	154 (51.16)	147 (48.84)
	Q22. Left ventricular ejection fraction (LVEF) ≥ 60% in children with congenital heart disease indicates normal cardiac function.	Cardiac function	198 (65.78)	103 (34.22)

**Table 4:** Responses of health personnel to case scenarios on screening checks for contraindications and precautions to vaccination (*N* = 301).

Statement	Theme	Correct n(%)	Incorrect n(%)
Q1. A child is in remission of bronchial asthma, and has been maintained with low-dose inhaled glucocorticoids for a long time. At present, the child is in good health, but has an allergy to milk. The recommendation for this child is that all types of vaccines can be given.	Asthma, food allergy	263 (87.38)	38 (12.62)
Q2. A child, who recently experienced an acute asthma attack and receiving oral glucocorticoids, is now exhibiting slight relief of symptoms. The recommendation for this child is to delay the administration of the vaccine until one month after discontinuation of drug use.	Asthmatic remission	241 (80.07)	60 (19.93)
Q3. A child diagnosed with severe combined immunodeficiency underwent successful stem cell transplantation and is currently undergoing a 3-month treatment for graft-versus-host disease. The recommendation for this child is to defer vaccination and resume it one year after cessation of graft-versus-host disease treatment.	Stem cell transplantation, combined immunodeficiency	208 (69.10)	93 (30.90)
Q4. For children experiencing infrequent febrile convulsions, defined as less than 3 seizures in six months and less than 4 seizures in a year, without a history of persistent convulsions lasting over half an hour, Inactivated vaccine is recommended after resolution of fever according to the immunization program. Live attenuated vaccine should be avoided. It is advised to administer one dose per vaccination.	Febrile convulsions	254 (84.39)	47 (15.61)
Q5. One year after allogeneic hematopoietic stem cell transplantation, the patient’s immune function returns to normal, and they can be vaccinated with various inactivated vaccines, irrespective of the use of immunosuppressive agents.	Hematopoietic stem cell transplantation	238 (79.07)	63 (20.93)
Q6. Children with IgA deficiency or specific polysaccharide antibody deficiency should be contraindicated for administration of live attenuated polio vaccine; however, they are eligible to receive hepatitis B vaccine, measles-rubella-mumps vaccine, and pertussis-diphtheria-tetanus vaccine.	IgA deficiency or specific polysaccharide antibody deficiency	210 (69.77)	91 (30.23)
Q7. Children with leukemia may receive inactivated vaccines six months after completing chemotherapy, while the administration of live attenuated vaccines can be considered following an evaluation of immune function one year post-chemotherapy.	Leukemia, chemotherapy	242 (80.40)	59 (19.60)
Q8. Patients with kidney disease in the asymptomatic phase, who are not receiving immunosuppressive agents, can receive various vaccines; while those on immunosuppressive agents with stable symptoms can be vaccinated with inactivated vaccines.	Kidney disease, immunosuppressant	191 (63.46)	110 (36.54)
Q9. If children born to HIV-infected mothers are diagnosed by medical institutions with AIDS-related or immunosuppressive symptoms, they should not receive live attenuated vaccines but rather inactivated vaccines.	Born to HIV-infected mothers	161 (53.49)	140 (46.51)
Q10. BCG vaccination is postponed for infants born to HIV-infected mothers until their HIV status is confirmed negative, after which they will receive a re-vaccination.	Born to HIV-infected mothers	173 (57.48)	128 (42.52)

## Data Availability

The data presented in this study is available on request from the corresponding author.

## Supplementary Materials

The following supporting information can be downloaded at: https://www.mdpi.com/article/10.3390/vaccines12060632/s1. Table S1. Questionnaire on perceptions and attitudes of healthcare professionals in vaccination clinics about screening for contraindications and precautions to vaccination; Table S2. Questionnaire on relevant knowledge and practical experience about contraindications and precautions to vaccination; Table S3. Sample characteristics in survey on perceptions and attitudes about screening for contraindications and precautions to vaccination (*N* = 761); Table S4. Sample characteristics in survey on relevant knowledge and practical experience about contraindications and precautions to vaccination (*N* = 301).
