# Supplementary material for: Knowledge and Attitudes about Contraindications and Precautions to Vaccination among Healthcare Professionals Working in Vaccination Clinics in Ningbo, China: A Cross-Sectional Survey
Source: Vaccines (Basel). 2024 Jun 6;12(6):632. doi: 10.3390/vaccines12060632 (PMC11209173; doi:10.3390/vaccines12060632)
Supplement: Supplementary file 1 [file vaccines-12-00632-s001.zip › vaccines-3022022-supplementary.pdf]

Table S1 Questionnaire on the perceptions and attitudes of healthcare professionals in vaccination clinics about screening for contraindications and precautions to vaccination

Part 1 Basic information

Q1 How old are you?
a. Aged 18 to 29
b. Aged 30 to 39
c. Aged 40 to 49
d. Aged 50 to 59

Q2 How many years have you worked as health personnel in vaccination clinics?
a. 0-4 years
b. 5-9 years
c. 10-19 years
d. 20+ years

Q3 What is your educational background?
a. College or below
b. Bachelor or above

Q4 What is your occupational specialty?
a. Clinical medicine
b. Public health
c. Nursing
d. Others

Q5 What is your professional title?
a. The senior professional titles
b. Intermediate professional titles
c. Primary professional titles
d. None

Q6 Are you full-time personnel in vaccination clinics responsible for screening for contraindications and precautions to vaccination?
a. Yes
b. No

Q7 How many times of training on contraindications and precautions to vaccination did you have?
a. Never
b. Once
c. Twice
d. 3 times or more

Part 2 Perceptions and attitudes about screening for contraindications and precautions to vaccination

Q8 Are you familiar with screening for contraindications and precautions to vaccination?
a. not familiar at all
b. not familiar
c. Neutral
d. familiar

e. very familiar

Q9 List the order of importance of screening for contraindications and precautions to vaccination in 4 steps of vaccination administration (screening for contraindications and precautions to vaccination, registration, vaccination, and observation after vaccination in clinics for 30 minutes).

a. First

b. Second

c. Third

d. Fourth

Q10 Score on a scale of 0-5 based on the following items of screening for contraindications and precautions to vaccination:

a. Degree of work stress: (   )

b. Degree of work difficulty: (   )

c. Time of screening for one case: (   )

d. Operation complexity of screening information system: (   )

e. Degree of work satisfaction: (   )

Table S2 Questionnaire on the relevant knowledge and practical experience on contraindications and precautions to vaccination

Part 1 Basic information

Q1 How old are you?

a. Aged 18 to 29

b. Aged 30 to 39

c. Aged 40 to 49

d. Aged 50 to 59

Q2 Have you had work experience of screening check for contraindications and precautions to vaccination?

a. Yes

b. No

Q3 What is your occupational speciality?

a. Clinical medicine

b. Public health

c. Nursing

d. Others

Q4 How many times of training on contraindications and precautions to vaccination have you had?

a. Never

b. Once

c. Twice

d. 3 times or more

Part 2 Knowledge and practical experience on contraindications and precautions to vaccination: Please pick "Yes" if it is correct, and pick "No" if it is wrong.

Part A Knowledge of contraindications and precautions to vaccination

Q5 The vaccine is contraindicated in children who are allergic to its components.

☐Yes; ☐No

Q6 Children with local reactions such as redness, swelling, heat and pain after vaccination are not contraindicated with this kind of vaccine.

☐Yes; ☐No

Q7 Children with fever after vaccination are not contraindicated with this vaccine.

☐Yes; ☐No

Q8 Vaccinations are not contraindicated in people with penicillin allergy.

☐Yes; ☐No

Q9 Attenuated live vaccines are not contraindicated in patients with complement deficiency.

☐Yes; ☐No

Q10 Vaccinations are not contraindicated in people with simple febrile seizures.

☐Yes; ☐No

Q11 Cerebral palsy is not a contraindication for vaccination.

☐Yes; ☐No

Q12 Children with physiological jaundice or breast milk jaundice can get vaccinated if they are in

good health.

☐Yes; ☐No

Q13 Children who have recovered from perianal abscess can be vaccinated with live attenuated polio vaccine.

☐Yes; ☐No

Q14 Individuals with mild to moderate iron deficiency anemia, in the absence of other symptoms, are eligible for vaccination.

☐Yes; ☐No

Q15 Children with mild to moderate hemolytic anemia, who do not exhibit acute hemolysis, are eligible for vaccination.

☐Yes; ☐No

Q16 Egg allergy is not a contraindication for measles-containing vaccines.

☐Yes; ☐No

Q17 Patients with mild to moderate hyperbilirubinemia in the context of chronic liver disease can be vaccinated by all types of vaccines.

☐Yes; ☐No

Q18 Asymptomatic children with congenital heart disease and preserved cardiac function can be vaccinated by all types of vaccines.

☐Yes; ☐No

Q19 Children with autoimmune diseases can be vaccinated with inactivated vaccines during their remission phase.

☐Yes; ☐No

Q20 Children treated with immunosuppressants can be vaccinated with inactivated vaccines.

☐Yes; ☐No

Q21 Patients with liver cirrhosis can be vaccinated with inactivated vaccines.

☐Yes; ☐No

Part B Knowledge of disease diagnosis and classification

Q22 Simple febrile seizures are characterized by generalized seizures lasting less than 15 minutes, which occur once during the course of a fever.

☐Yes; ☐No

Q23 Stable seizure control refers to epilepsy patients who have been seizure-free for at least 6 months, regardless of their use of antiepileptic drugs.

☐Yes; ☐No

Q24 The category of primary immunodeficiency includes chronic granulomatous disease, combined immunodeficiency, and immune dysregulation disease; however, thrombocytopenic purpura does not fall within this classification.

☐Yes; ☐No

Q25 Type 1 diabetes, dermatomyositis, Sjogren's syndrome, and ankylosing spondylitis are all classified as autoimmune disorders.

☐Yes; ☐No

Q26 Left ventricular ejection fraction (LVEF) $\geqslant$ 60% in children with congenital heart disease indicates normal cardiac function.

☐Yes; ☐No

Part C Case scenario

Q27 A child is in remission of bronchial asthma, and has been maintained with low-dose inhaled glucocorticoids for a long time. At present, the child is in good health, but has an allergy to milk. The recommendation for this child is that all types of vaccines can be given.

☐Yes; ☐No

Q28 A child, who recently experienced an acute asthma attack and receiving oral glucocorticoids, is now exhibiting slight relief of symptoms. The recommendation for this child is to delay the administration of the vaccine until one month after discontinuation of drug use.

☐Yes; ☐No

Q29 A child diagnosed with severe combined immunodeficiency underwent successful stem cell transplantation and is currently undergoing a 3-month treatment for graft-versus-host disease. The recommendation for this child is to defer vaccination and resume it one year after cessation of graft-versus-host disease treatment.

☐Yes; ☐No

Q30 For children experiencing infrequent febrile convulsions, defined as less than 3 seizures in six months and less than 4 seizures in a year, without a history of persistent convulsions lasting over half an hour, the administration of inactivated vaccine is recommended following resolution of fever according to the immunization program. Live attenuated vaccine should be avoided. It is advised to administer one dose per vaccination.

☐Yes; ☐No

Q31 One year after allogeneic hematopoietic stem cell transplantation, the patient's immune function returns to normal, and they can be vaccinated with various inactivated vaccines, irrespective of the use of immunosuppressive agents.

☐Yes; ☐No

Q32 Children with IgA deficiency or specific polysaccharide antibody deficiency should be contraindicated for administration of live attenuated polio vaccine; however, they are eligible to receive hepatitis B vaccine, measles-rubella-mumps vaccine, and pertussis-diphtheria-tetanus vaccine.

☐Yes; ☐No

Q33 Children with leukemia may receive inactivated vaccines six months after completing chemotherapy, while the administration of live attenuated vaccines can be considered following an evaluation of immune function one year post-chemotherapy.

☐Yes; ☐No

Q34 Patients with kidney disease in the asymptomatic phase, who are not receiving immunosuppressive agents, can receive various vaccines; while those on immunosuppressive agents with stable symptoms can be vaccinated with inactivated vaccines.

☐Yes; ☐No

Q35 If children born to HIV-infected mothers are diagnosed by medical institutions with AIDS-related or immunosuppressive symptoms, they should not receive live attenuated vaccines but rather inactivated vaccines.

☐Yes; ☐No

Q36 BCG vaccination is postponed for infants born to HIV-infected mothers until their HIV status is confirmed negative, after which they will receive a re-vaccination.

☐Yes; ☐No

Table S3 Sample characteristics in the survey on the perceptions and attitudes regarding screening for contraindications and precautions to vaccination (N=761)

Characteristics	N	Percentage(%)
Total	761	100
Age		
18-29	148	19.45
30-39	347	45.60
40-49	233	30.62
50-59	33	4.34
Job experience		
0-4 years	108	14.19
5-9 years	114	14.98
10-19 years	359	47.17
20+ years	180	23.65
Education		
College or below	135	17.74
Bachelor or above	626	82.26
Occupational specialty		
Clinical medicine	127	16.69
Public health	195	25.62
Nursing	404	53.09
Others	35	4.60
Professional title		
Senior	68	8.94
Intermediate	341	44.81
Primary	307	40.34
None	45	5.91
Full-time personnel for screening for contraindications and precautions to vaccination		
Yes	259	34.03
No	502	65.97
Occurrences of training on contraindications and precautions to vaccination		
Never	37	4.86
Once	318	41.79
Twice	248	32.59
3 times or more	158	20.76

Table S4 Sample characteristics in the survey on the relevant knowledge and practical experience on contraindications and precautions to vaccination (N=301)

Characteristics	N	Percentage(%)
Total	301	100
Age		
18-29	69	22.92
30-39	141	46.84
40-49	80	26.58
50-59	11	3.65
Occupational specialty		
Clinical medicine	21	6.98
Public health	128	42.52
Nursing	144	47.84
Others	8	2.66
Work experience in screening for contraindications and precautions to vaccination		
Yes	250	83.06
No	51	16.94
Occurrences of training on contraindications and precautions to vaccination		
Never	16	5.32
Once	59	19.60
Twice	80	26.58
3 times or more	146	48.50
